# Coagulation factor XII protease domain crystal structure

**DOI:** 10.1111/jth.12849

**Published:** 2015-03-11

**Authors:** M Pathak, P Wilmann, J Awford, C Li, BK Hamad, PM Fischer, I Dreveny, LV Dekker, J Emsley

**Affiliations:** Centre for Biomolecular Sciences, School of Pharmacy, University of NottinghamNottingham, UK

**Keywords:** active site, catalytic domain, factor XII, plasma prokallikrein, tertiary protein structure, zymogens

## Abstract

**Background:**

Coagulation factor XII is a serine protease that is important for kinin generation and blood coagulation, cleaving the substrates plasma kallikrein and FXI.

**Objective:**

To investigate FXII zymogen activation and substrate recognition by determining the crystal structure of the FXII protease domain.

**Methods and results:**

A series of recombinant FXII protease constructs were characterized by measurement of cleavage of chromogenic peptide and plasma kallikrein protein substrates. This revealed that the FXII protease construct spanning the light chain has unexpectedly weak proteolytic activity compared to β-FXIIa, which has an additional nine amino acid remnant of the heavy chain present. Consistent with these data, the crystal structure of the light chain protease reveals a zymogen conformation for active site residues Gly193 and Ser195, where the oxyanion hole is absent. The Asp194 side chain salt bridge to Arg73 constitutes an atypical conformation of the 70-loop. In one crystal form, the S1 pocket loops are partially flexible, which is typical of a zymogen. In a second crystal form of the deglycosylated light chain, the S1 pocket loops are ordered, and a short α-helix in the 180-loop of the structure results in an enlarged and distorted S1 pocket with a buried conformation of Asp189, which is critical for P1 Arg substrate recognition. The FXII structures define patches of negative charge surrounding the active site cleft that may be critical for interactions with inhibitors and substrates.

**Conclusions:**

These data provide the first structural basis for understanding FXII substrate recognition and zymogen activation.

## Introduction

FXII is a central component of the contact system, which also includes the serine proteinase prekallikrein (PK) and the non-enzymatic cofactor high molecular weight kininogen [1]. FXII circulates in the blood as an 80-kDa single-chain polypeptide zymogen with no detectable enzymatic activity. The contact system can be activated by diverse negatively charged polymers, including kaolin [1], nucleic acids [2], and collagen [3]. It has also been recently demonstrated that neutrophil extracellular traps, which form networks of fibers primarily composed of DNA, can activate the contact system in a process linked to innate immunity, the generation of antimicrobial peptides, and complement activation [4].

The FXII Arg353–Val354 peptide bond is cleaved by kallikrein, generating α-FXIIa, which has a heavy chain of 50 kDa, connected to a light chain of 28 kDa by the Cys340–Cys467 disulfide bridge. Once a small amount of α-FXIIa is generated, this cleaves PK to generate kallikrein, which then mediates efficient cleavage of further FXII in a feedback loop that amplifies production of α-FXIIa and kallikrein. Subsequent cleavage of α-FXIIa results in loss of the heavy chain and generation of the isolated protease domain termed β-FXIIa, which contains only a nine amino acid peptide heavy chain remnant disulfide bonded to the protease domain [1].

Human genome-wide studies have reported associations of single-nucleotide polymorphisms in FXII with thrombosis and a shortening of the activated partial thromboplastin time [5]. FXII knockout mice are protected against thrombosis and ischemic stroke in models of the disease [6]. It has been proposed that targeting of FXII could result in medicines with a safer anticoagulation profile than the currently available anticoagulants, such as warfarin [7]. A recently described antibody, 3F7, which binds specifically to the FXII protease domain forms the basis of a new anticoagulation therapy [8].

**Fig 1:**
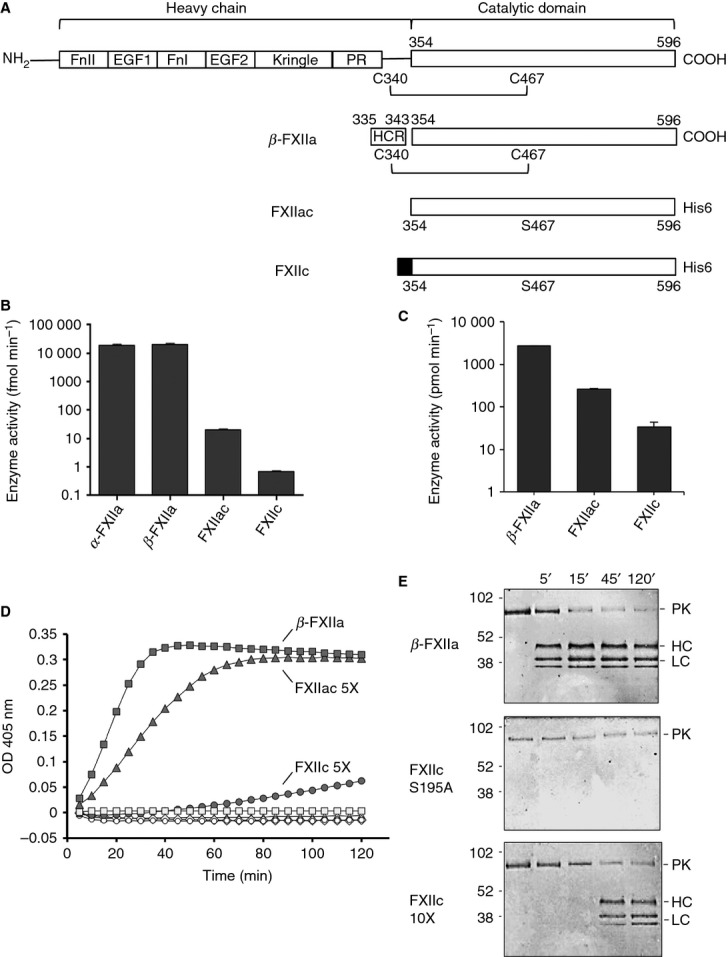
FXII domain boundaries and constructs. (A) The domain structure of FXII is shown at the top (FnII, Fibronectin type II domain; EGF, epidermal growth factor-like domain; FnI, fibronectin type I domain; PR, Pro-rich region), and illustrated below are the constructs FXIIc and FXIIac used in this study, with the unpaired Cys467 mutated to Ser highlighted. The heavy chain (HC) remnant (HCR) resulting from autoproteolysis of FXII is boxed for β-FXIIa. The shaded region at the N-terminus of FXIIc represents the N-terminal cap of two residues, Arg and Ser. (B) Enzymatic activities of the recombinant proteases FXIIc and FXIIac (1.5 μm) are illustrated, as assessed with the S2302 peptide substrate cleavage assay, as compared with commercial α-FXIIa (0.0253 μm) and β-FXIIa (0.0333 μm). The scale on the *y*-axis is logarithmic. (C) The rate of enzymatic activity of FXII proteases measured in the prekallikrein (PK) cleavage experiments shown in (D) is presented with a scale on the *y*-axis that is logarithmic. (D) Native substrate PK (50 nm) was incubated in reaction buffer containing S2302 peptide substrate, in the presence of β-FXIIa (▪) (1 nm), FXIIac (▴) (5 nm), and FXIIc (•) (5 nm). Cleavage of S2302 was monitored by following changes in OD_405 nm_. PK alone (◊) and FXII samples at the concentration used do not cleave S2302 at any appreciable rate in the absence of PK (○, □, ▵). (E) Specific proteolytic cleavage of substrate PK is shown for FXIIc and β-FXIIa. Anti-human PK polyclonal antibody western blots of SDS-PAGE are shown for a time course of PK proteolysis in the presence of β-FXIIa protease (top gel), a 10× concentration of the FXIIc active site mutant Ser195Ala (middle gel), and FXIIc (bottom gel). Lanes 1–5 represent 0–120 min. The arrows to the right of each blot indicate the zymogen PK and the HC and two light chains (LCs) of cleaved PK. The positions of molecular mass standards (kDa) are shown on the left of the figure.

FXII has evolved from duplication of the hepatocyte growth factor activator (HGFA) gene ancestor. Both have a similar domain organization, which consists of an N-terminal fibronectin type II domain (FnII) followed by an epidermal growth factor-like (EGF)1 domain, fibronectin type I (FnI), an EGF2 domain, a Kringle domain, and a C-terminal serine protease domain with an additional Pro-rich (PR) region unique to FXII (Fig. 1A). A crystal structure has been described for the FXII fibronectin type I domain and EGF2 domain, but no structure exists for the protease [9]. Here, we describe the first crystal structures of the FXII protease domain, which reveals a zymogen-like conformation.

## Materials and methods

### Cloning, expression, and purification

A gene fragment encoding human FXII light chain residues Val354–Ser596 (mature protein sequence numbering) was cloned into the pMT puro vector for expression with the DES system (Invitrogen, Carlsbad, CA, USA) [10]. The Cys467Ser point mutation was introduced by overlap PCR mutagenesis to remove an unpaired Cys that could potentially cause aggregation. At the N-terminus, the signal sequence corresponds to a *Drosophila* homolog of the immunoglobulin-binding chaperone protein secretion signal, and at the C-terminus a polyhistidine tag sequence HHTGTRHHHHHH was added. Use of the *Bgl*II restriction site resulted in two additional residues, Arg and Ser, at the N-terminus of the FXII sequence, and this sample was termed FXIIc. This restriction site was deleted with overlap PCR mutagenesis to generate the native catalytic domain FXIIac (a = active; c = catalytic domain). *Drosophila* S2 cells were grown in Dulbecco's modified Eagle's medium supplemented with 10% fetal bovine seum at 28 °C, and transfection was performed with calcium phosphate. Cells were grown for an additional 48 h before selection with puromycin to establish stable cell lines. Serum-free Express Five insect culture medium (Invitrogen), containing secreted proteins, was collected, and 30–85% (NH_4_)_2_SO_4_ fractionation resulted in a protein pellet; further purification was performed with Ni–sepharose column affinity chromatography and gel filtration chromatography. N-terminal sequencing of the purified samples confirmed the removal of the signal peptide and that the correct sequence was present at the N-terminus. Deglycosylation with PNGase F (NEB, Hitchin, UK) was carried out for 24 h at 30 °C in 50 mm sodium phosphate (pH 7.4).

### Crystallization and structure determination

Purified samples of FXIIac and FXIIc were dialyzed into 20 mm Tris-HCl (pH 7.4) and 100 mm NaCl, and concentrated to 17 mg mL^−1^. Crystallization was performed at 19 °C and 10 °C with sparse matrix screens (Qiagen, Hilden, Germany; Molecular Dimensions, Newmarket, UK) in sitting drop plates. Crystals were observed for glycosylated FXIIac in conditions of 0.1 m HEPES (pH 7.5), 1.6 m (NH_4_)_2_SO_4_, and 2% (w/v) poly(ethylene glycol) 1000 in the presence of PPACK at 10 °C. Deglycosylated FXIIc grew from solutions containing 1.2 m (NH_4_)_2_SO_4_, 0.05 m trisodium citrate, and 3% isopropanol. Single crystals were transferred to the reservoir solution containing 25% glycerol, and flash cooled in liquid nitrogen. Diffraction data were collected at DIAMOND beamline I04, at 2.4 Å for FXIIac and 2.1 Å for FXIIc. Data were processed and reduced with xds [11] and the ccp4 suite in space groups P3_2_21 (FXIIac) and P4_1_2_1_2 (FXIIc). The structures were determined by molecular replacement (phaser) with coordinates from the HGFA protease domain (Protein Data Bank [PDB]: 1YC0). Both models were built with coot [12] and refined with refmac (Table 1).

**Table 1:** Data collection and refinement statistics

	FXIIc	FXIIac
Data collection		
Space group	P4_1_2_1_2	P3_2_21
Cell dimensions		
*a*, *b*, *c* (Å)	124.1, 124.1, 38.2	137.1, 137.1, 37.0
*α*, *β*, *γ* (°)	90, 90, 90	90, 90, 120
Wavelength (Å)	0.9763	0.97949
Resolution (Å)	2.1	2.4
*R*_merge_ *	0.085 (0.378)	0.069 (0.816)
*I*/σ*I*	23.0 (1.71)	11.0 (2.3)
Completeness (%)	95.2 (84.3)	98.2 (98.6)
Multiplicity	12.9	5.6
CC 1/2†	1.00 (0.98)	1.00 (0.76)
Unique reflections	16 114	15 737
Refinement‡		
*R*_work_	0.222 (0.279)	0.218 (0.397)
*R*_free_	0.262 (0.278)	0.295 (0.417)
Overall *B*-factor (A^2^)	29.2	73.6
Stereochemical r.m.s.d.		
Bond lengths (Å)	0.013	0.016
Bond angles (°)	1.86	1.96
Ramachandran plot		
Most favored (%)	93.3	85.3
Allowed (%)	6.7	14.7
Outliers (%)	0	0

r.m.s.d., root mean square deviation. *Values in parentheses correspond to the highest-resolution shell.

* *R*_merge_ = Σ_*h*_ Σ_*i*_|*I*_*i*_(h) − <*I*(*h*)>/|Σ_*h*_Σ_*i*_*I*_*i*_(*h*), where *I* is the observed intensity and <*I*> is the average intensity of multiple observations from symmetry-related reflections calculated with xds.

† Correlation coefficient value calculated with xds to determine the resolution cutoff.

‡ All values were calculated with refmac. *R*_work_ = Σ_*h*_||*F*_o_|*h* − |*F*_c_|*h*|/Σ_*h*_|*F*_o_|*h*, where *F*_o_ and *F*_c_ are the observed and calculated structure factors, respectively. *R*_free_ was computed as for *R*_work_, but only for (5%) randomly selected reflections, which were omitted from refinement.

### Assays of FXII activity

Amidolytic activity was measured with the chromogenic substrate H-d-Pro-Phe-Arg-*p*-nitroaniline, termed S2302 (Chromogenix, Epsom, UK) [13]. FXIIc and FXIIac (final protein concentration: 1.5 μm), β-FXIIa (0.0333 μm) and α-FXIIa (0.0253 μm) were assayed at 37 °C in a final volume of 100 μL of 0.01 m phosphate buffer (0.0027 m potassium chloride and 0.137 m sodium chloride, pH 7.4). Initial rates were determined with 2 mm S2302 substrate by measuring the release of *p*-nitroaniline at 405 nm.

In the assay measuring PK conversion to kallikrein β-FXIIa (1 nm), FXIIc (5 nm) and FXIIac (5 nm) were incubated with PK (50 nm) in reaction buffer containing 200 μm S2302 (Chromogenix) at 37 °C protein concentration. Changes in OD_405 nm_ reflecting conversion of PK to α-kallikrein were monitored with a microplate reader.

For kinetic experiments, the fluorogenic substrate Pro-Phe-Arg-7-AMC (P9273; Sigma, Gillingham, UK) was used to measure enzymatic activity in a 30-μL final volume of phosphate-buffered saline (pH 7.3) (BR0014G; Oxoid, Basingstoke, UK) supplemented with 0.03% Tween-20. The reaction was started by addition of enzyme to the substrate, after which release of the fluorescent group was monitored every 5 min for 5 h by excitation at 380 nm and emission at 460 nm. The enzyme activity rates at constant enzyme–substrate concentration were determined from the time course and used to derive the kinetic parameters. Data were fitted in graphpad prism, with the non-linear regression Michaelis–Menten algorithm (Table 2).

**Table 2:** Enzyme kinetic parameters for factor XII

Sample	*k*_cat_(s^−1^)	*K*_m_ (μm)	*k*_cat_/*K*_m_ (L mol^−1^ s^−1^)
α-FXIIa	0.67 ± 0.01	222 ± 3	2991
β-FXIIa	0.76 ± 0.01	260 ± 8	2904
FXIIac	0.67 × 10^−3^ ± 0.01 × 10^−3^	195 ± 4	3
FXIIc	2.90 × 10^−5^ ± 0.09 × 10^−5^	283 ± 13	0.1
FXIIc S195A	< 0.01 × 10^−3^	ND	ND

ND, not determined. Enzymatic activity was measured as described. Values are given as reported, with the standard error of the regression analysis calculated with graphpad prism (non-linear regression with the Michaelis–Menten equation).

### Mass spectrometry

Nano-electrospray ionization mass spectra were collected on a Waters SYNAPT instrument (Elstree, UK) with a quadruple time-of-flight mass analyzer calibrated with horse heart myoglobin. Samples were prepared by desalting FXIIc with a C4 ZipTip into 80 : 20 MeCN/H_2_O and 0.1% trifluoroacetic acid. Subsequent mass spectra were acquired over a 500–5000 *m* *z*^−1^ range in positive ion mode. Minimal smoothing and noise reduction of spectra were applied to the raw data.

## Results

### Recombinant FXII light chain catalytic activity

The recombinant proteins investigated were FXIIac, which has the native N-terminus, and a zymogen-like protease FXIIc, in which the N-terminus is blocked by the presence of two additional amino acids, Arg and Ser. SDS-PAGE of the purified proteins is shown in Fig. S1, and N-terminal sequencing was performed, confirming the expected residues at the N-terminus and proper removal of the secretory signal sequence (data not shown). The Asn74 N-linked glycan in the recombinant FXIIc was characterized by mass spectrometry, which revealed masses corresponding to two species that were either 2GlcNac + 4Man or 2GlcNac + Fuc (Fig. S1). Commercially available β-FXIIa represents an autoproteolytic end-product resulting from incubation of plasma-purified FXII zymogen with dextran sulfate (Enzyme Research Laboratories, Swansea, UK). β-FXIIa contains a nine amino acid remnant peptide sequence NGPLSCGQR spanning residues 335–343 termed the heavy chain remnant.

To characterize the catalytic activity of the two recombinant FXII proteins, we measured the hydrolysis of chromogenic peptide substrate S2302 in comparison with α-FXIIa and β-FXIIa by using published methodology [14]. The FXIIac protease catalyzed cleavage of S2302, whereas the zymogen-like protease FXIIc had ∼ 10-fold lower measurable activity (Fig.1B). Unexpectedly, a comparison of FXIIc with commercial plasma-purified α-FXIIa and β-FXIIa revealed a ∼ 1000-fold greater rate of substrate cleavage for the latter (Fig.1B). This experiment was repeated with a second fluorescent peptide substrate, Pro-Phe-Arg-7-AMC (Sigma) [14]; this confirmed these results, and measured values of *k*_cat_/*K*_m_ are shown in Table 2. To verify this observation, we utilized a third assay, which involves cleavage of the native substrate PK by the FXII protease samples and measurement of enzymatic activity with the resulting activated PK. The results from this indirect assay revealed a reduced rate of ∼ 10-fold less cleavage for FXIIac than for β-FXIIa, and ∼ 10-fold less for FXIIc than for FXIIac (Fig.1C). Control experiments in this assay with PK or FXII alone did not result in measurable substrate cleavage (Fig.1D). To examine the cleavage of PK by our FXII samples, we used a time course monitored with SDS-PAGE, and, as expected, addition of FXIIc and β-FXIIa to PK resulted in specific cleavage, generating the characteristic fragmentation resulting in bands for the PK heavy (single band) and light (double band) chains (Fig.1E).

The notable difference between the S2302/7-AMC peptide substrate and PK cleavage experiments probably reflects the different requirements for the enzyme to cleave the tripeptide sequence of S2302 and to cleave the large 80-kDa substrate of PK, which may undergo additional interactions with the FXII protease. Overall, these data reveal that the native FXIIac light chain has 10-fold reduced catalytic activity in a PK cleavage assay as compared with β-FXIIa, and a 1000-fold reduced activity cleaving tripeptide substrates.

### The FXIIac crystal structure

**Fig 2:**
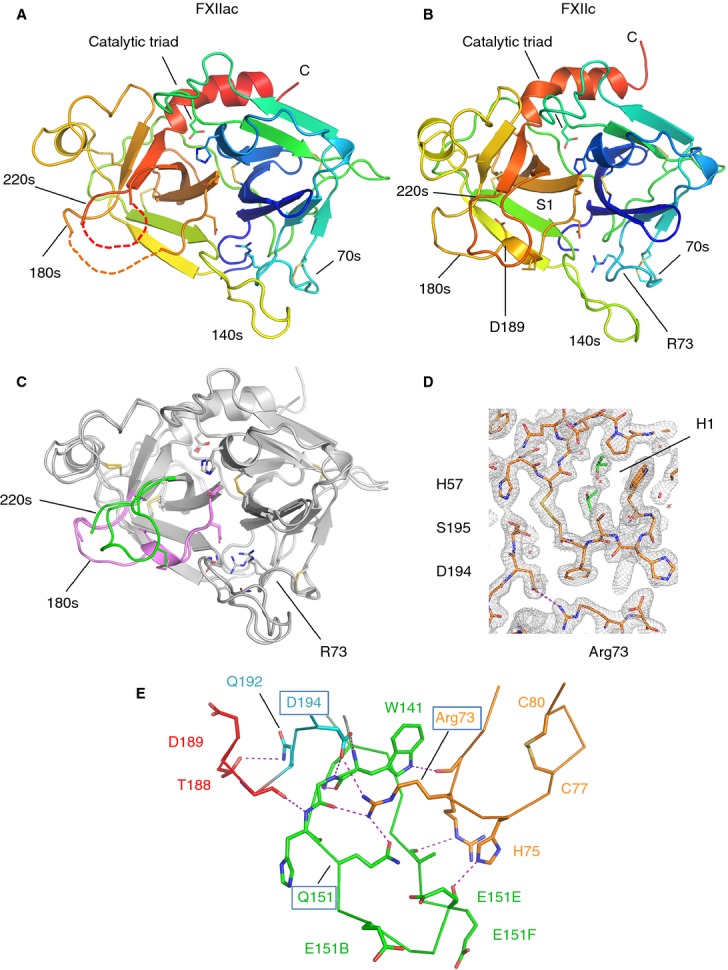
FXII protease structures. (A) Cartoon diagram of the FXIIac topology colored from the N-terminus to the C-terminus, with the N-terminal β-barrel in blue/green and the C-terminal β-barrel in yellow/orange. Loops containing residue ranges derived from the chymotrypsin sequence numbering are labeled 70s, 140s, 180s, and 220s. Disulfide bonds are shown in yellow. Regions not visible in the electron density are shown as dotted lines. (B) Cartoon diagram of the FXIIc structure. (C) FXIIac and FXIIc crystal structures superposed. The two structures are represented as cartoon traces with the 180-loop colored pink and the 220-loop colored green. (D) Electron density map (2*F*_o_ – *F*_c_ coefficients) for the final refined coordinates of FXIIc at 2.1-Å resolution shown in the area of the active site residue Ser195 contoured at 1.5 root mean square deviation. Isopropanol molecules bound in the H1 pocket are shown in green as sticks, and water molecules as red crosses. (E) Interactions between loops from the two FXIIc β-barrels are shown, illustrating how Arg73 connects to residues from both the 180-loop and 140-loop. The 80-loop, 140-loop and 220-loop are colored orange, green, and cyan, respectively, with S1 helix residues in red. Electrostatic and hydrogen-bonding interactions are shown as purple dotted lines. The positively charged residues Arg72, Arg73 and His75 from the 70-loop are shown as sticks in orange.

Using the recombinant FXIIac protease in crystallization experiments, we followed a similar approach as the FXI protease (FXIac) [15] of cocrystallization with the inhibitors PCK, PPACK, and corn trypsin inhibitor. FXIIac crystallized both in the presence and in the absence of PPACK at 10 °C, and the FXIIac crystal structure was determined by molecular replacement with the structure of the homologous HGFA protease and refined to an *R*-factor of 0.219 (data collection and refinement statistics are summarized in Table 1). The topology is shown in Fig.2A, with the characteristic double β-barrel fold with the catalytic triad of His57, Asp102 and Ser195 shown. The nomenclature of chymotrypsin residue numbering is used throughout with FXII mature residue numbering, and the corresponding FXII immature residue numbers are listed in Table S1 for key residues. There are six disulfide bridges in the FXIIc structure, three of which are well conserved among other chymotrypsin-like proteases (Cys42–Cys58, Cys168–Cys182, and Cys191–Cys220), two (Cys50–Cys111 and Cys136–Cys201) of which occur in HGFA [16] and tissue-type plasminogen activator (t-PA) [17], and one of which (Cys77–Cys80) is unique to FXII (Fig. S2). Two GlcNAc residues could be observed in the electron density, and were modeled covalently linked to Asn74, where they undergo loose packing interactions with the N-terminal β-barrel.

A key feature that identifies the FXIIac structure as a zymogen is the absence of an oxyanion hole (Gly193 and Ser195) and a buried zymogen-like conformation of the Asp194 side chain, whereby it forms hydrogen bonds with the main chain nitrogen of Trp141. A second feature is that residues 182–183 from the 180-loop and residues 217–221 from the 220-loop of the S1 pocket are not visible in the electron density. We have previously observed in the FXI zymogen crystal structure, where there are no crystal packing contacts in the area of the active site, that the 180-loop and 220-loop are also not present in the electron density, suggesting S1 loop flexibility [18]. The FXIIac zymogen conformation was unexpected, but is in agreement with the enzyme kinetic data described above, which showed that FXIIac has reduced catalytic activity as compared with β-FXIIa. The N-terminus is not observed in the FXIIac electron density, and Val16–Leu23 are presumed to be flexible, and do not efficiently insert into the core of the protease domain in this context (see discussion).

### FXIIc crystal structure

FXIIc is a protein-engineered zymogen-like construct with two additional residues present at the N-terminus, resulting from the cloning strategy, that effectively block the native N-terminal residue Val354. We term this the zymogen-like construct, as it is similar to the result of protein engineering of the N-terminus of FXa to induce zymogenicity [19]. FXIIc crystallized more readily than FXIIac under a range of conditions for both glycosylated and deglycosylated samples at 19 °C. Data were collected to 2.1-Å resolution for the deglycosylated FXIIc sample (Table 1), and Fig.2D shows the electron density in the region of the active site residue Ser195.

An additional feature observed in FXIIc but not in FXIIac is that the S1 pocket structure is clearly defined and all residues from the 180-loop and 220-loop are observed, with coiling of residues 189–194 into a single turn of an α-helix (colored pink in Fig.2C). In FXIIc, Arg73 extends its side chain guanidinium group from the 70-loop of the N-terminal β-barrel to form a direct salt bridge with the buried Asp194 side chain (Fig.2D). The Arg73 side chain from the 70-loop is stabilized by a number of interactions with the 140-loop, resulting in encirclement of the Arg73 guanidinium group and the formation of hydrogen bonds via the side chain of Gln151 (Fig.2E). The conformation of the S34 pocket is maintained in FXIIac and FXIIc, but the region of the H1 pocket is altered, with a 90° rotation of the Trp35 side chain and a shift in the main chain, resulting in a repositioning of this residue such that it now forms a lid on top of the H1 pocket and becomes partially buried.

### A catalytically incompetent conformation of the FXIIac and FXIIc active site

**Fig 3:**
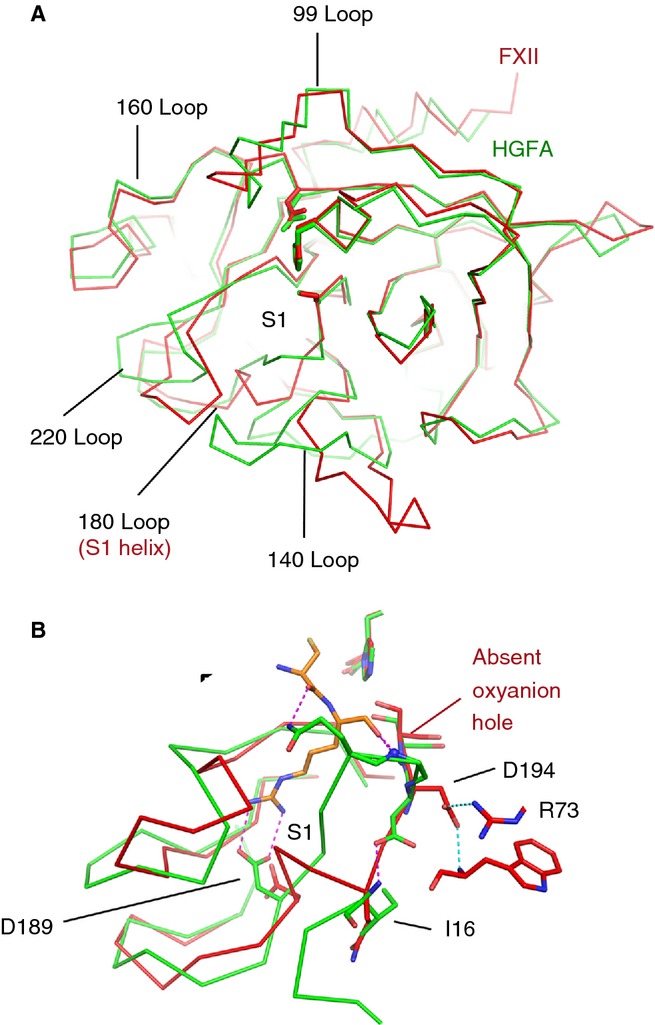
FXIIc structural comparisons with the activated protease hepatocyte growth factor activator (HGFA). (A) C-α traces are shown for superposed protease domains FXIIc (red) and HGFA (green). (B) Close-up view of the superposition in the area of the S1 pocket shows key side chains highlighted as sticks. The HGFA S1 pocket structure illustrates the conformation of an activated protease bound to inhibitor (Kunitz domain, KD1) shown in orange (only two residues from KD1 are shown, and interactions are shown as dotted purple lines. Main chain nitrogen atoms from the HGFA oxyanion hole are shown as blue spheres.

We performed comparisons with the FXIIc structure and the activated Ser protease domain of HGFA, which is the closest homolog to FXII in amino acid identity [20]. Superposition of FXIIc with the HGFA catalytic domain resulted in 222 equivalent residues with an overall root mean square deviation (r.m.s.d.) of 1.8 Å (47% amino acid identity; dali [21]). The C-α traces of the superposed structures are illustrated in Fig.3A. Residues from the catalytic triad and the N-terminal β-barrel superimpose well, whereas large differences are observed in the C-terminal β-barrel and loop-140, loop-180, and loop-220. The largest difference observed is a 25-Å change for the tip of the 140-loop; a smaller 14-Å change is observed for the 220-loop. In HGFA, the 140-loop forms a hairpin-like structure folded up against the side of the C-terminal β-barrel, interacting with the 180-loop close to the entrance of the S1 pocket [22]. This folded-up 140-loop conformation is commonly observed in activated proteases, and contrasts with FXIIc, in which the 140-loop extends away from the body of the C-terminal β-barrel.

The FXIIc S1 pocket structure superposed with the HGFA protease structure, which has a Kunitz domain (KD)1 inhibitor bound [23], is shown in Fig.3B. In the HGFA–KD1 complex, the side chain groups of Asp189 and Gln192 engage the substrate P1 Arg side chain and P2 main chain, respectively, and the P1 Arg main chain carbonyl contacts the oxyanion hole main chain nitrogens [16,23]. There are three areas of major conformational difference between the HGFA and FXIIc structures that confirm that FXIIc is a catalytically incompetent zymogen structure: (i) the peptide bond of Asp194 is flipped by 180°, resulting in the absence of the oxyanion hole; (ii) Asp189 is folded back in the S1 pocket, and undergoes a water-mediated interaction with the side chain of Tyr228 (Fig.3B); and (iii) the largest change affects the side chain of Gln192, which is displaced by 12 Å, forming a hydrogen bond with the side chain of Thr188 in FXIIc. Asn223 from the adjacent loop forms an α-helix-capping hydrogen bond with the main chain nitrogen of Ala190. All residues that contact the inhibitor in HGFA are in different positions in FXIIc, by virtue of the concerted coiling of residues 189–194 into the FXII S1 α-helix. These three differences in the 180-loop and 140-loop further characterize the FXIIc structure as having a zymogen conformation.

### FXIIc surface pockets and charge distribution

**Fig 4:**
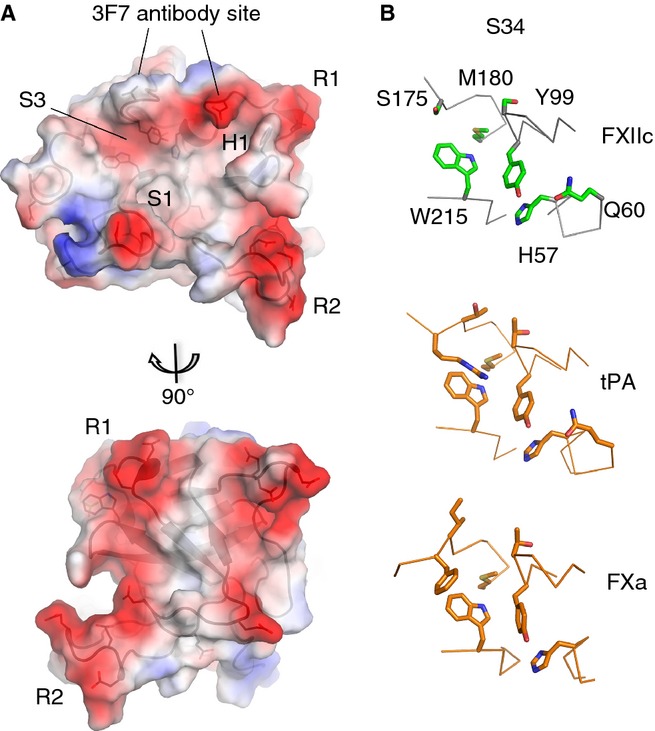
FXIIc surface pockets and charge. (A) Charge surface representation of the FXIIc crystal structure (negative = red and positive = blue). Ridges of negative charge are labeled R1 and R2. S1, S34 and H1 pockets are labeled. Two views related by a 90° rotation are shown. (B) Zoom-in view of the S34 pocket structure for FXIIc (top), with side chains shown as sticks (green) and equivalent residues shown for tissue-type plasminogen activator (t-PA) (middle) and FXa (bottom) colored orange.

The FXII protease amino acid sequence is acidic, with a calculated pI of 5.2. An electrostatic potential surface representation of the area around the active site loops reveals clusters of negatively charged residues organized into two patches, which encircle the substrate-binding pockets labeled R1 (Asp60A, Glu62, and Asp63) and R2 (Glu151B, Glu151E, and Glu151F) in Fig.4A. The two patches surround the active site, and a 90° rotation of the surface in Fig.4A shows that the negative charge extends down the exterior face of the N-terminal β-barrel. Interestingly, a sequence alignment with related proteases reveals that the negatively charged patch R1 also occurs in urokinase plasminogen activator (u-Pa), whereas R2 only occurs in FXII (Fig. S3). This overall charge character contrasts with thrombin, which has positively charged anion-binding exosites (overall calculated pI of 8.8 for the thrombin protease sequence). The 3F7 antibody, which binds specifically to the FXII protease domain and forms the basis of a new anticoagulation therapy [8], has been mapped as binding to Asp60 from patch R1 and Ile101 from the area of the S34 pocket, and thus probably functions by sterically blocking substrate binding to FXI and PK (Fig.4A).

The H1 pocket is a distinctive feature that is located in the center of the N-terminal β-barrel, with the side chains of Leu33, Leu64, Leu59, Leu106 and Trp35 contributing to the hydrophobic character, and is positioned directly in front of the S1 pocket in the C-terminal β-barrel (Figs2D and 4A). The shape of the S1, S2, S3 and S4 pockets is of special interest, as coagulation protease substrates and inhibitors commonly interact here [24]. The area of the S34 pocket is formed by the 220-loop, 170-loop, and 99-loop, with Trp215 being a constant feature of this pocket, occurring in all of the coagulation proteases [16] and FXII. The FXIIc S34 pocket is shown in Fig.4B, with Trp215 and Tyr99 highlighted. Superposition of FXIIc with the crystal structures of the homologous u-PA (42% identity, r.m.s.d. 2.2 Å, 225 equivalent residues, PDB 3MHW) and t-PA (42% identity, r.m.s.d. 2.3 Å, 225 equivalent residues, PDB 1RTF) reveals that u-PA also has an insertion in the 99-loop, whereas t-PA has the closest similarity, with the same 99-loop length as FXII. t-PA also has the Tyr99 present in FXII, and the loop structures are closely superimposable. Examination of other coagulation factors reveals that the more distantly related FXa also has this arrangement, and Fig.4B shows that Met180 is present as a constant feature in all three of these proteases, contributing to the bottom of the S34 pocket.

## Discussion

In the current study, we report the first crystal structures of the FXII protease domain light chain. Both FXIIac and protein-engineered zymogen-like construct FXIIc structures reveal the conformational hallmarks of a zymogen, owing to the lack of an oxyanion hole, a disordered (FXIIac) or helical (FXIIc) 180-loop, and an extended 140-loop. The disordered portions of the 180-loop and 220-loop in the FXIIac structure are consistent with nuclear magnetic resonance studies on thrombin, which has a similar S1 pocket architecture to FXII, showing that these loops are disordered in solution in the absence of substrate [25].

**Fig 5:**
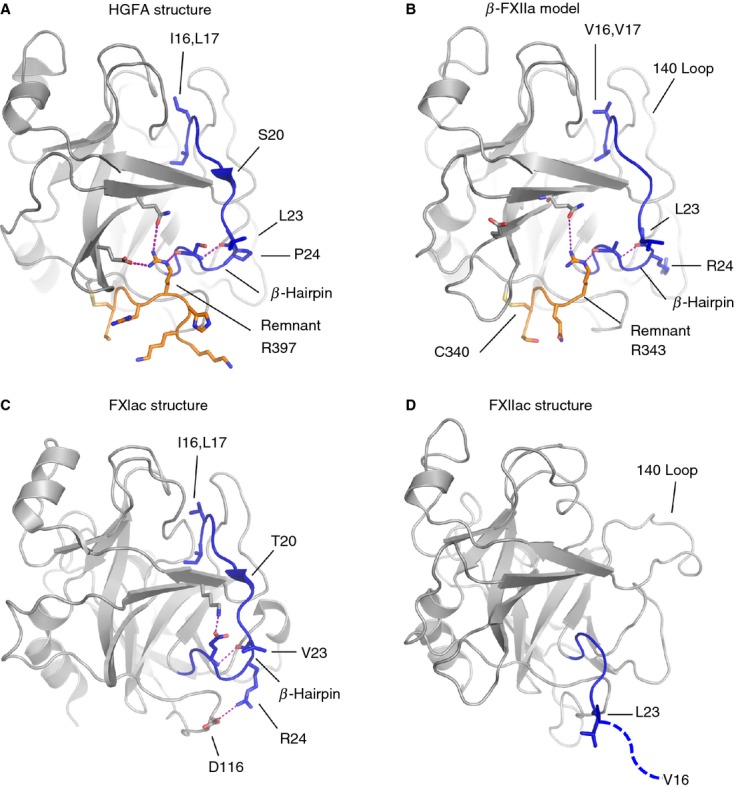
Cartoon diagrams showing stabilizing interactions in the region of the heavy chain remnant (orange) and the activation loop (blue) are compared for different proteases with salt bridges or hydrogen bonds illustrated as a dotted purple line. (A) Hepatocyte growth factor activator (HGFA) (Protein Data Bank [PDB]: 1YC0), (B) β-FXIIa, homology model, (C) FXIa (PDB: 1XX9), which has no remnant, and (D) the FXIIac structure reported here. The FXIIac activation loop does not have a formed β-hairpin, and residues after Leu23 are not present in the electron density (dotted blue line).

The zymogen conformation of FXIIac is unexpected, as protease constructs for u-Pa, FXIa and trypsin lacking a heavy chain remnant have been crystallized in the active conformation [15,26,27]. To determine a structural basis for this difference, we generated a homology model of β-FXIIa by using the HGFA structure (program swissmodel [28]). Figure5A shows the HGFA crystal structure with the activation loop (blue) and remnant (orange) highlighted, and the model of β-FXIIa is shown in Fig.5B. Eight amino acids from the HGFA remnant are present in the crystal structure (PDB: 1YC0), which only partially overlaps with the remnant present in β-FXIIa, which is proteolyzed further to Arg343. One feature observed in HGFA and the majority of the trypsin-like class of protease structures is a β-hairpin in the C-terminal region of the activation loop involving residues 23–26. In HGFA, direct interactions are observed between the remnant and this β-hairpin, whereby the Arg397 side chain has a hydrogen-bonding interaction with the carbonyl group of Ser26. In the homology model of β-FXIIa, Arg343 is equivalent to Arg397, and is modeled undergoing a similar interaction with the base of the activation loop.

A structural basis for the low catalytic activity and the zymogen-like conformation of FXIIac could be that a direct interaction between the remnant and the amino acids of the activation loop is required to form the β-hairpin, and thus for Val16 to be positioned correctly to insert into the protease core and form the S1 pocket. Clearly, not all proteases have this requirement, as some are produced without the remnant, such as FXIa shown in Fig.5C. Here, it is notable that, unlike in FXIIa, the FXIa activation loop forms two salt bridges from Glu26 and Arg24 to residues in the protease domain [15]. Evidence of stabilizing interactions in the region of the β-hairpin is also observed in the trypsin structure, where no remnant is present, and the β-hairpin conformation is fixed by a disulfide bond from Cys22 to Cys157. These types of stabilizing interaction are absent in the model of β-FXIIa.

**Fig 6:**
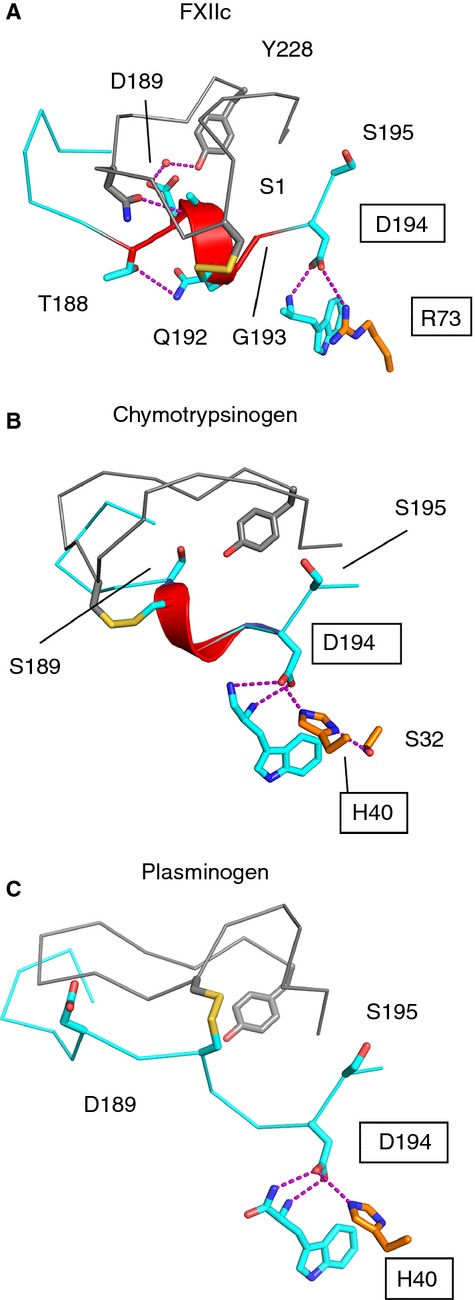
FXIIc S1 pocket comparison with chymotrypsinogen and plasminogen zymogen crystal structures. Cartoon diagrams of the S1 pocket 180-loop and 220-loop showing a similar structure for (A) FXIIc, (B) chymotrypsinogen (Protein Data Bank [PDB]: 2CGA), shown with zymogen triad residues His40, Ser32, Asp194 as sticks, and (C) plasminogen (PDB: 4DUU). The S1 helix is colored red, and zymogen triad residues are boxed.

A novel structural feature that we observe in FXIIac and FXIIc is that the 70-loop has an unusual conformation, whereby Arg73 extends to form a salt bridge with the side chain of Asp194. The engagement of the negative Asp194 carboxyl group is a key switch between the activated and inactive forms of chymotrypsin-like proteases. In the zymogen crystal structures of chymotrypsinogen, FXI, trypsin, and plasminogen [29], an Asp194–His40 side chain interaction is observed. His40 is not present in FXII, whereas the side chain of Arg73 occupies a similar position to His40 (Fig.6). Further structural studies on the full-length FXII zymogen coupled with mutagenesis experiments will be required to verify whether Arg73 plays a role in stabilizing the FXII zymogen and whether the remnant is required to stabilize the activated form in FXIIa.

The negatively charged amino acid clusters surrounding the S1 pocket define surface patches that distinguish FXII from other coagulation proteases, and could potentially interact with substrates and inhibitors. This could complement the overall net positive charge of the substrate, FXI, implying that charge complementary is important for efficient FXII substrate recognition. These features contrast with those of thrombin, which has well-characterized positively charged exosites on the surface of the protease domain. Both thrombin and FXIIa can activate FXI, but thrombin cleavage has a greater dependence on the addition of polyanions than FXIIa [30].

Targeting coagulation factor serine protease active sites with inhibitors is the subject of intensive research, and studies with antibodies that bind FXII have suggested that this may provide an effective form of anticoagulation therapy [8,31]. Overall, the data presented here provide novel and important contributions to our understanding of the structure of the FXII protease zymogen conformation, and provide an initial basis for understanding both substrate and inhibitor binding and zymogen activation.

## Addendum

M. Pathak, P. Wilmann, J. Awford, B. Hamad and C. Li, designed the research, performed experiments, analyzed and interpreted data. P. Fischer, I. Dreveny, L. Dekker and J. Emsley designed the research, analyzed and interpreted data, and wrote the manuscript.

## Supporting Information

**Fig. S1.** Biochemical characterization of the recombinant FXII protease domain.

**Fig. S2.** Cartoon diagram of the FXIIc disulfide bonds.

**Fig. S3.** FXII protease domain sequence alignment with related proteases.

**Table S1.** Residues in the chymotrypsin and FXII numbering systems.
