# Supplementary material for: Coagulation factor XII protease domain crystal structure
Source: J Thromb Haemost. 2015 Mar 11;13(4):580–91. doi: 10.1111/jth.12849 (PMC4418343; doi:10.1111/jth.12849)
Supplement: Supplementary file 1 — Fig. S1. Biochemical characterization of the recombinant FXII protease domain. [file jth0013-0580-sd1.pdf]

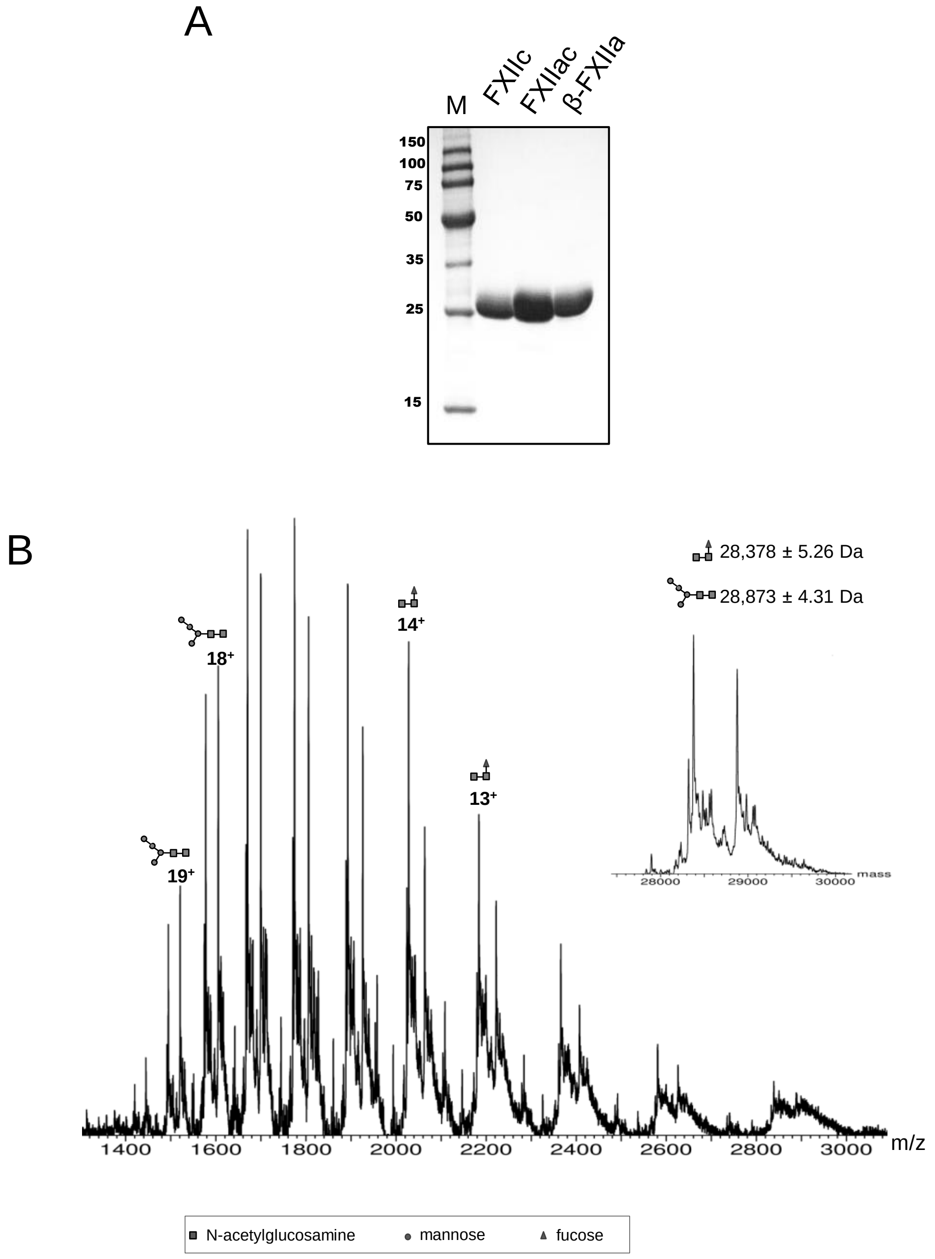

Fig. S1. Biochemical characterisation of recombinant FXII protease domains. (A) Coomassie stained reducing SDS PAGE of purified proteins recombinant FXIIc, FXIIac and commercial β-FXIIa. (B) Nano-ESI mass spectrum of the FXIIc construct sprayed from 80:20 MeCN:H2O + 0.1% TFA. Samples were buffer exchanged in 50mM ammonium acetate. Protein concentrations of 100μM were used. The raw spectrum of all observed charge states is shown, with deconvoluted masses for each species detected is highlighted. A transformed spectrum is shown on the right with the relative abundance of the detected species. The spectrum shows two species of mass 28873 Da and 28378 Da compared to a theoretical mass of 27844 Da for FXIIc. A theoretical mass of FXIIc containing 2 GlcNAc molecules and 4 mannose molecules is 28899.0 Da and 2GlcNaC and 1 Fucose molecule is 28396.77 Da.
