# Supplementary material for: Coagulation factor XII protease domain crystal structure
Source: J Thromb Haemost. 2015 Mar 11;13(4):580–91. doi: 10.1111/jth.12849 (PMC4418343; doi:10.1111/jth.12849)
Supplement: Supplementary file 2 — Fig. S2. Cartoon diagram of the FXIIc disulfide bonds. [file jth0013-0580-sd2.pdf]

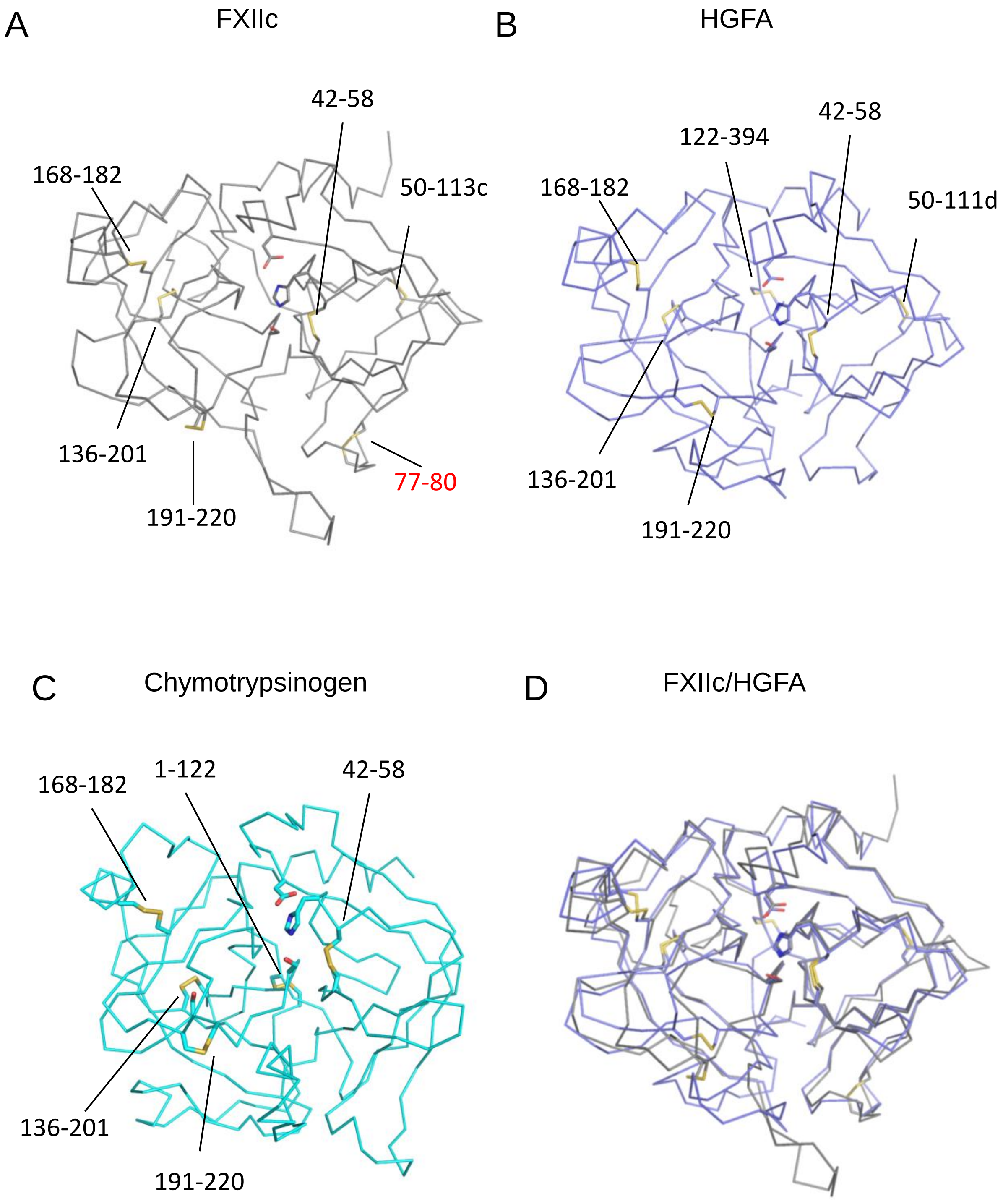

Fig. S2. FXIIc disulphide bonds and structural comparisons with the protease HGFA and chymotrypsinogen. C-α traces are displayed for (A) FXIIc, (B) HGFA protease (pdb:1YC0), (C) chymotrypsinogen (pdb:2CGA) and (D) FXIIc (grey) and HGFA (purple) superposed. Disulphide bonds are colored yellow. Catalutic triad residues are illustrated as sticks in each case. Labelled in red is the disulphide bond unique to FXII.
