# Supplementary material for: Coagulation factor XII protease domain crystal structure
Source: J Thromb Haemost. 2015 Mar 11;13(4):580–91. doi: 10.1111/jth.12849 (PMC4418343; doi:10.1111/jth.12849)
Supplement: Supplementary file 3 — Fig. S3. FXII protease domain sequence alignment with related proteases. [file jth0013-0580-sd3.pdf]

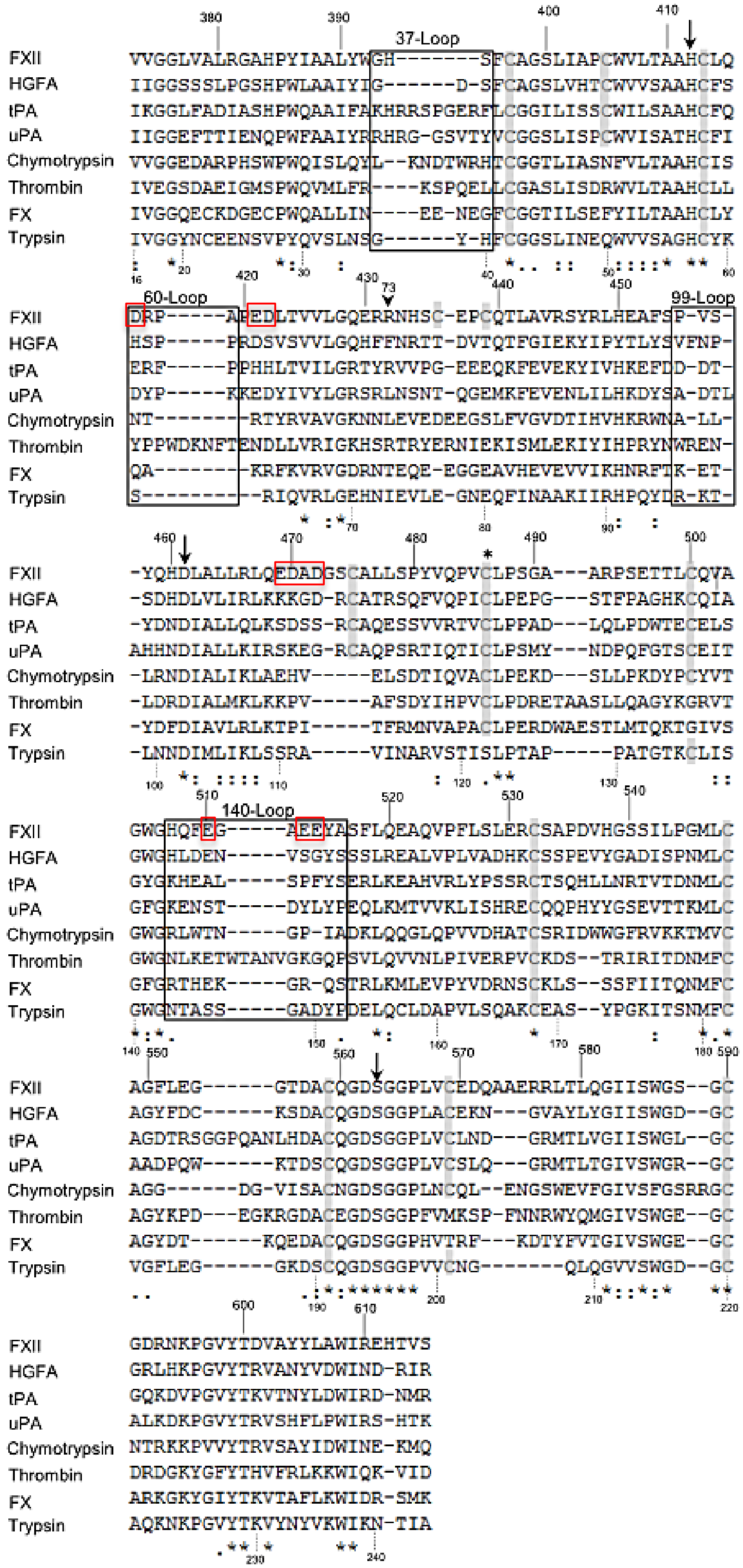

Fig. S3. FXII protease domain sequence alignment with related proteases. Amino acid sequences of chymotrypsin-like protease domains homologous to FXII are aligned. Native sequential residue numbering for FXII appears above the sequences and chymotrypsinogen numbering appears below. Selected surface loops are shown as boxed regions and labelled. Arrows indicate the catalytic triad. Residues boxed in red contribute to surface charged negative patches illustrated in Figure 4A with R1 corresponding to the 60-loop and R2 the 140-loop.
