# Supplementary material for: Coagulation factor XII protease domain crystal structure
Source: J Thromb Haemost. 2015 Mar 11;13(4):580–91. doi: 10.1111/jth.12849 (PMC4418343; doi:10.1111/jth.12849)
Supplement: Supplementary file 4 — Table S1. Residues in the chymotrypsin and FXII numbering systems. [file jth0013-0580-sd4.docx]

**Table S1.** Key residues in the chymotrypsin and FXII numbering systems.

| **FXII** | **Chymotrypsin** | **FXII** | **Chymotrypsin** |
| --- | --- | --- | --- |
| Ala389 | Ala32 | Gly504 | Gly140 |
| Leu390 | Leu33 | Trp505 | Trp141 |
| Ser395 | Ser40 | Ala516 | Ala152 |
| Phe396 | Phe41 | Cys532 | Cys168 |
| His 412 | His57 | Ser540 | Ser174 |
| Leu414 | Leu59 | Met546 | Met180 |
| Glu421 | Glu62 | Thr556 | Thr188 |
| Asp422 | Asp63 | Asp557 | Asp189 |
| Leu423 | Leu64 | Ala558 | Ala190 |
| Arg431 | Arg72 | Gln560 | Gln192 |
| Arg432 | Arg73 | Asp562 | Asp194 |
| Asn433 | Asn74 | Ser563 | Ser195 |
| His434 | His75 | Trp586 | Trp215 |
| Tyr458 | Tyr99 | Gly591 | Gly220 |
| Leu465 | Leu106 | Asp592 | Asp221 |
| Val502 | Val138 | Asn594 | Asn223 |

Sequence numbering shown for FXII is the polypeptide with the signal sequence which can be converted to the FXII mature protein numbering by subtraction of the 19 amino acid signal peptide.
